# Supplementary figures and images for: Creatinine clearance is key to solving the enigma of sex difference in in-hospital mortality after STEMI: Propensity score matching and mediation analysis
Source: PLoS One. 2023 May 3;18(5):e0284668. doi: 10.1371/journal.pone.0284668 (PMC10155957; doi:10.1371/journal.pone.0284668)

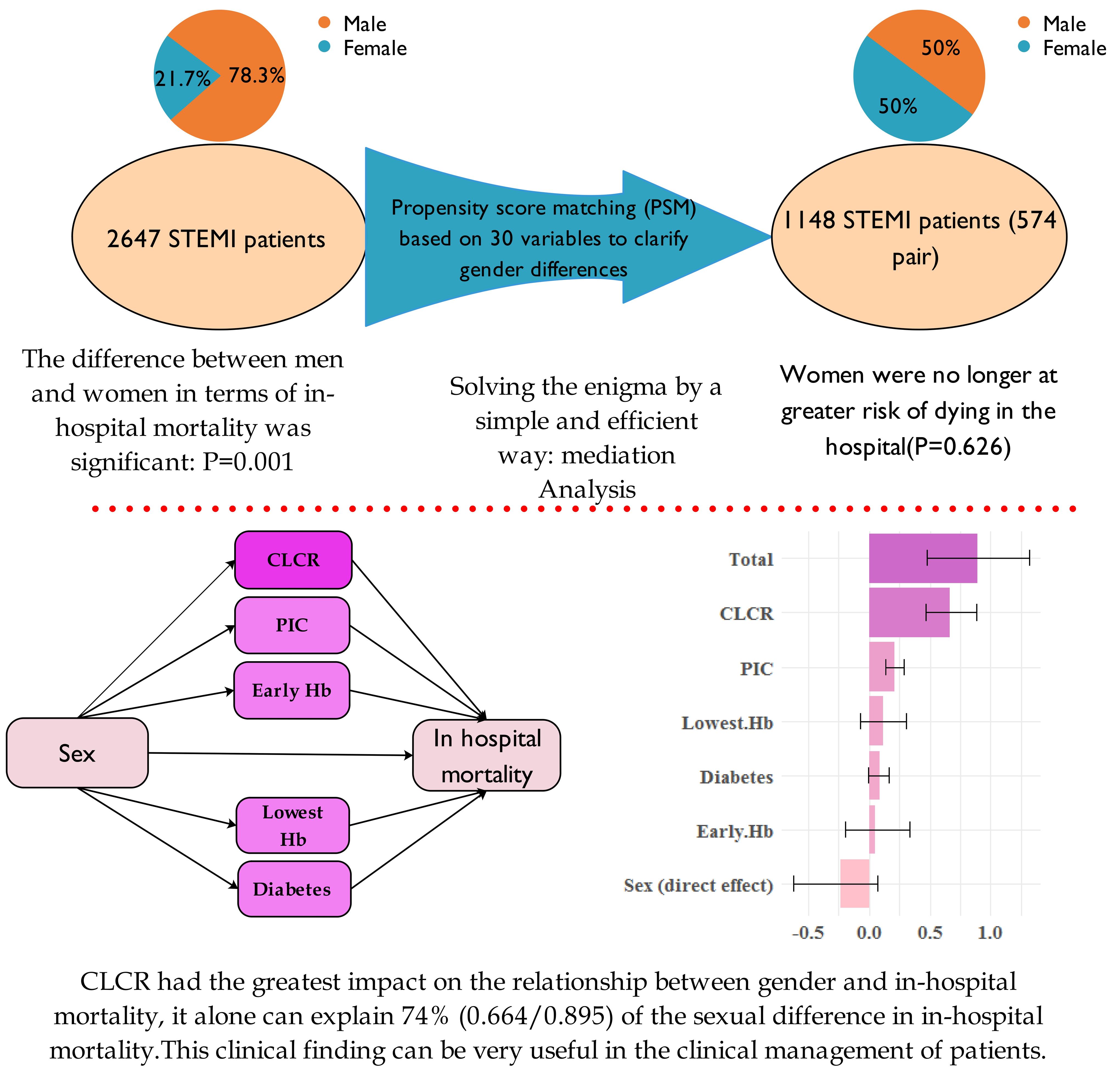

Supplement: S1 Graphical abstract — (TIF) [file pone.0284668.s004.tif]
