# Supplementary material for: Creatinine clearance is key to solving the enigma of sex difference in in-hospital mortality after STEMI: Propensity score matching and mediation analysis
Source: PLoS One. 2023 May 3;18(5):e0284668. doi: 10.1371/journal.pone.0284668 (PMC10155957; doi:10.1371/journal.pone.0284668)
Supplement: Attachment — Submitted filename: Response to Reviewers.docx [file pone.0284668.s005.docx]

Journal Requirements:

When submitting your revision, we need you to address these additional requirements.

Dear editor,

Thanks for your comments and suggestions, we tried to apply all your requests to the manuscript.

1. Please ensure that your manuscript meets PLOS ONE's style requirements, including those for file naming. The PLOS ONE style templates can be found at <https://journals.plos.org/plosone/s/file?id=wjVg/PLOSOne_formatting_sample_main_body.pdf> and
   <https://journals.plos.org/plosone/s/file?id=ba62/PLOSOne_formatting_sample_title_authors_affiliations.pdf>

Answer:

Thanks, corrections were done.

2. Please provide additional details regarding participant consent. In the ethics statement in the Methods and online submission information, please ensure that you have specified what type you obtained (for instance, written or verbal, and if verbal, how it was documented and witnessed). If your study included minors, state whether you obtained consent from parents or guardians. If the need for consent was waived by the ethics committee, please include this information.

Answer:

Thanks, corrections were done.

Reviewers' comments:

Reviewer #1: Major

Dear reviewer,

Thanks for your comments and suggestions, we tried to apply all your requests in "Track-change" mode to the manuscript and highlighted all corrections.

Abstract
The abstract does not adequately summarise the content of the main text. In particular, the method section should be summarised accurately.

Answer:

Thanks for your attention, corrections were done.

Introduction
The aim is to investigate the association between gender and hospital deaths due to STEMI. The statistical methods and other information are described, but not enough is said about the background to the analysis on mediating effects.

Answer:

Thanks, corrections were done, we hope it will be satisfactory.
Discussion
The CLCR has not been examined for confounding with other influencing factors besides gender, such as weight and Scr values. This point should be mentioned.

Answer:

Thanks so much, the aim of our research is to elucidate the causal chain between gender (exposure/treatment) and in-hospital mortality (outcome), based on all studied variables that CLCR is one of them, as we did not address this issue. So that, CLCR effect on the causal chain between sex and in-hospital mortality was evaluated together with other suspected intermediate variables. Therefore, drawing connections outside this domain is very difficult and ambiguous. Hence, we request from you waive this case if possible.

Minor
The first sentence on page 9 is duplicated.
Answer: Thanks a lot, the duplicate sentence was removed.
Figure and Tables
Abbreviations or units are not mentioned. Vertical lines in the tables should be removed to make them easier to read.

Answer:

Thanks, we added abbreviations and corresponding units for all variables in tables 1–3, where possible, plus appropriate abbreviations were added to figure legends, and vertical lines in tables were removed.

Many of the abbreviations are not explained in the text either and should be reviewed in their entirety (such as LBBB, ECG in page 3).

Answer:

Thanks for your attention, corrections were done, for each abbreviation, we used its full equivalent.
